# A nuclear sensor of mitochondrial function

**DOI:** 10.18632/oncotarget.4687

**Published:** 2015-06-27

**Authors:** Richard M. Monaghan, Gino B. Poulin, Alan J. Whitmarsh

**Affiliations:** Faculty of Life Sciences, University of Manchester, Manchester, UK

**Keywords:** Chromosome Section, mitochondria, retrograde signalling, reactive oxygen species, CLK-1

Mitochondria are organelles that host many metabolic processes, including the generation of energy in the form of adenosine triphosphate (ATP) through oxidative phosphorylation. They contain their own genome, however, the vast majority of mitochondrial proteins are encoded in the nucleus. Nuclei sense changes in mitochondrial functioning via retrograde signaling pathways, resulting in modified gene expression to restore homeostasis. These pathways can be triggered by altered ATP synthesis, increased levels of unfolded or damaged proteins, or changes in the production of reactive oxygen species (ROS), a major by-product of the electron transport chain. Indeed, inhibition of electron transport activity leads to enhanced ROS levels and, somewhat paradoxically, promotes increased lifespan in a range of model organisms. The complex relationship between mitochondrial function, ROS and aging has been a topic of intense research for many years, with particular focus on the retrograde signaling pathways that act as the conduit of information between mitochondria and nuclei [1].

**Figure 1:**
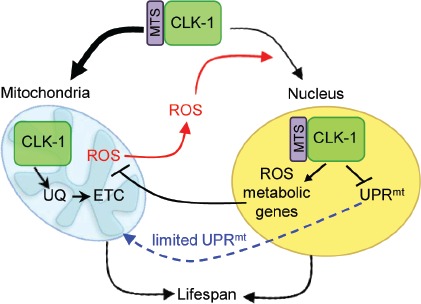
Nuclear CLK-1 regulates mitochondrial homeostasis and longevity The majority of CLK-1 localizes to mitochondria via a mitochondrial targeting sequence (MTS), where it is required for ubiquinone (UQ) biosynthesis, an essential cofactor in the electron transport chain (ETC). A pool of CLK-1 is redirected to nuclei by a ROS-dependent pathway responsive to changes in oxidative metabolism. In the nucleus, CLK-1 regulates the expression of retrograde genes that promote ROS metabolism and limit the activation of the UPRmt. Both mitochondrial and nuclear functions of CLK-1 contribute to regulating lifespan.

We have uncovered a novel nuclear role for the respiratory enzyme CLK-1 in maintaining mitochondrial function by regulating the expression of genes that promote ROS homeostasis and dampen the activity of a key stress pathway, the mitochondrial unfolded response (UPR^mt^) [2]. Importantly, CLK-1 localizes to nuclei dependent upon mitochondrial ROS production, indicating that it acts as a barometer of respiratory activity that can directly mediate retrograde signaling to the nucleus (Figure 1). CLK-1 (also called COQ7) is a diiron containing monooxygenase that catalyzes the hydroxylation of 5-demethoxyubiquinone, a critical step in the biosynthesis of the mitochondrial electron transport chain cofactor ubiquinone. *C. elegans clk-1* null mutants and heterozygous mice display defective oxidative phosphorylation, increased ROS levels and, similar to many other respiration mutants, have extended lifespans [3]. It had been assumed that this longevity phenotype was solely due to CLK-1 acting in mitochondria. However, the expression of a CLK-1 mutant that specifically localizes to nuclei partially suppressed the extended lifespan of *clk-1* null *C. elegans,* indicating that the nuclear form of CLK- 1 independently affects longevity [2]. As elevated ROS levels and activation of the UPR^mt^ are reported to promote longevity, their suppression by nuclear CLK-1 would be consistent with its role in limiting lifespan.

We propose that CLK-1 functions as a rheostat to maintain ROS homeostasis and keep stress-responsive pathways in check. Elevated ROS produced by mitochondria direct a pool of CLK-1 to the nucleus where it regulates the expression of genes that decrease ROS levels and limit activation of the UPR^mt^. Conversely, decreased ROS lead to a reduction in nuclear CLK-1 and relieve its effects on gene expression allowing ROS levels to recover and the UPRmt to return to basal levels, thus maintaining homeostasis (Figure 1). CLK-1 therefore coordinates ROS responses with the UPR^mt^, a pathway that protects against the accumulation of unfolded or misfolded proteins within mitochondria. This suggests that distinct mitochondrial signals can be integrated at the level of nuclear gene expression to provide a unified response.

Important questions concerning the dual mitochondrial and nuclear functions of CLK-1 remain unanswered. Firstly, how do changes in ROS levels lead to the nuclear accumulation of CLK-1? The mitochondrial and nuclear forms of CLK-1 are distinguishable; the mitochondrial form is cleaved at the N-terminus by a protease following import while the nuclear form is the full-length protein, indicating that it has not been imported into mitochondria prior to its translocation to the nucleus. Interestingly, mutation of the mitochondrial targeting sequence in CLK-1 results in its nuclear accumulation independently of ROS levels [2]. This would be consistent with a mechanism by which ROS inhibit mitochondrial import of CLK-1, but whether this occurs by blocking mitochondrial targeting of CLK-1 or by preventing import through either the outer or inner mitochondrial membranes is unclear. Interestingly, a transcription factor component of the *C. elegans* UPR^mt^, the pathway suppressed by nuclear CLK-1, may be similarly regulated. ATFS-1 is targeted to mitochondria and rapidly degraded in the absence of UPRmt induction, while activation of the UPR^mt^ leads to impaired import of newly synthesized ATFS-1 and its redirection to the nucleus where it regulates the expression of genes that restore mitochondrial proteostasis [4]. Thus, the inhibition of mitochondrial import in response to changes in mitochondrial function may be a common mechanism of regulating retrograde signaling to the nucleus.

A second unresolved issue is the mechanism by which CLK-1 regulates gene expression. This is independent of its role in ubiquinone biosynthesis [2]. CLK-1 is a relatively small protein (around 20kDa) that does not contain obvious elements associated with DNA binding or transcriptional activation/suppression. However, it associates with many genomic loci suggesting that it may directly regulate transcription [2]. An interesting possibility is that the enzymatic activity of CLK-1 modifies transcriptional regulatory factors or chromatin.

There is precedent for mitochondrial enzymes regulating nuclear gene expression, including the yeast arginine biosynthesis enzyme Arg5,6 [5] and the pyruvate dehydrogenase complex [6]. Furthermore, many mitochondrial proteins have been detected in nuclei although their roles remain uncharacterized [7]. Our study provides a novel example of how distinct forms of a respiratory enzyme can be separately targeted to mitochondria or nuclei to regulate different cellular processes. This research contributes to our understanding of the diverse signaling mechanisms controlling mitochondrial function and ROS homeostasis that can affect aging and disease.
